# Monoclonal antibodies specific for *Bacteroides fragilis* enterotoxins BFT1 and BFT2 and their use in immunoassays

**DOI:** 10.1371/journal.pone.0173128

**Published:** 2017-03-03

**Authors:** Saraspadee Mootien, Paul M. Kaplan

**Affiliations:** L2 Diagnostics LLC, New Haven, Connecticut, United States of America; Duke University School of Medicine, UNITED STATES

## Abstract

We have developed 22 mouse IgG_1_ monoclonal antibodies (mAbs) against *Bacteroides fragilis* zinc metalloprotease toxins 1 and 2 (BFT1 and BFT2). Mice were immunized with recombinant BFT1 or BFT2 proteins with metalloprotease activity. Eight of the mAbs bind specifically to BFT1. One mAb, 2H6, binds specifically to BFT2. The remaining 13 mAbs bind to both BFT1 and BFT2. The eight BFT1-specific mAbs recognize at least five different epitopes on the toxin. Four of the BFT1-specific mAbs neutralized rBFT1 metalloprotease activity. Only one of these four mAbs, 1D9, neutralizes the cytotoxic effect of BFT1. Here, we describe the development of enzyme-linked immunosorbent assays (ELISAs) to detect BFT1 or BFT2 toxin in an isotype-specific manner. The sandwich ELISAs have a detection limit of 20 to 40 ng/ml when purified recombinant BFT protein is diluted into PBS. The sandwich ELISA can be used to distinguish and quantify levels of rBFT1 and rBFT2 in stool. This ELISA can be an important tool to investigate the association between BFT expression by enterotoxigenic *B*. *fragilis* and diseases such as diarrhea, inflammatory bowel disease and colorectal cancer.

## Introduction

*Bacteroides fragilis* (*B*. *fragilis*) is a commensal, Gram negative, non-motile, non-spore forming gut bacterium, and is one of the most abundant anaerobes found in humans. It is the most common anaerobic organism isolated from clinical stool specimens, bloodstream infections, and abdominal abscesses. *B*. *fragilis* strains that secrete a metalloprotease toxin are termed Enterotoxigenic *Bacteroides fragilis* (ETBF), while the non-toxin secreting strains are called nontoxigenic *B*. *fragilis* (NTBF). The protease toxin that ETBF secretes is a potent zinc-dependent metalloprotease toxin called the *B*. *fragilis* toxin (BFT, encoded by the *bft* gene) [1]. Three known variants of the *bft* gene have been identified to date: *bft*-1, *bft*-2 and *bft*-3 [2–4]. About 90% of ETBF strains isolated from stool specimens to date are either *bft-1* or *bft-2* [3]. ETBF was first identified when it was shown to be associated with diarrhea in lambs [5], and it is now considered to be an emerging pathogen associated with human diarrheal diseases worldwide in both adults and children [6–12]. The three known isotypes of BFT all cleave the tumor suppressor and intercellular adhesion protein, E-cadherin [13, 14]. Cleavage of E-cadherin can result in increased mucosal permeability, and leads to mucosal immune system exposure to luminal bacterial antigens and presumably mucosal inflammation. Reports show that ETBF can induce a serious inflammatory diarrhea resembling shigellosis in human populations [15, 16].

As well as diarrheal diseases, ETBF has been implicated in Inflammatory Bowel Disease (IBD) and colorectal cancer (CRC). ETBF expression of BFT can induce colitis in C57Bl/6 mice [9]. ETBF has been shown to induce chronic inflammation and CRC in Min mice [10]. Although NTBF is also capable of colonizing Min mice, it does not induce inflammation and does not lead to CRC. Studies of various human populations from the past decade have indicated links between ETBF colonization and active IBD [17, 18], colitis, and colorectal cancer (CRC) [10, 19], and more recent studies have improved the association [20, 21]. However, a significant portion of asymptomatic populations (4 to 30%) are colonized with ETBF [1]. Association studies with large cohort populations may reveal ETBF colonization conditions that can lead to disease. Such studies have been hampered by the lack of rapid, sensitive and standardized assays capable of detecting BFT in clinical samples in an isotype-specific fashion. The current methods for detection of ETBF are lengthy or require a high level of laboratory skill and expensive materials. The most commonly used definitive assay relies on culture of stool samples on Bacteroides Bile Esculin (BBE) agar under anaerobic conditions, followed by PCR for identification of the *bft* gene, and even this method does not reliably identify the ETBF isotypes without sequencing the PCR products. The ability to diagnose exposure to *Helicobacter pylori* (Hp), either by detection of specific serum anti-Hp antibodies or detection of gastric or fecal Hp was critical to linking Hp to peptic ulcer disease and gastric cancer [22–25]. A similar capability for detection of ETBF, and BFT isotypes, may yield important advances to explain both the etiology of ETBF-associated bowel diseases, including CRC, and the basis for the significant asymptomatic population of ETBF patients.

Here, we describe the development of two highly specific ELISAs for the detection of the secreted *B*. *fragilis* zinc-metalloprotease toxin isotypes, BFT1 and BFT2. It is hoped that development of separate ELISAs for each isotype will provide diagnostic tools with superior ability to discriminate the pathology of each. These ELISAs are more sensitive than a previously described ELISA [26] that does not differentiate between BFT isotypes. In this study, we characterize a group of mouse mAbs developed after immunization of mice with recombinant BFT zinc metalloprotease. The specificities of these newly developed mAbs were assessed using ELISA protocols and BFT cytotoxicity neutralization assays. We also discuss the relevance of these mAbs to the development of diagnostics tools for both research and clinical purposes. The ELISA assays demonstrate isotype-specific detection of rBFT in human stool samples.

## Materials and methods

### Cloning, expression and purification of recombinant BFT1 (rBFT1) and recombinant BFT2 (rBFT2)

Profragilysin-1 (pro-BFT1; UnitProt Q9S5W0) and profragilysin-2 (prot-BFT2; UniProt O05091) DNAs were chemically synthesized by GenScript and cloned into pET28a expression vector at the NdeI-XhoI site with an N-terminal His-tag. The 1194 bp genes encoded both the prodomain (PD) and mature catalytic domain (CD). The inserts were codon-optimized for *Escherichia coli* (*E*. *coli*). The sequences of all constructs were verified by DNA sequencing. For protein expression, the plasmids were transformed into *E*. *coli* Origami-2 (DE3) cells (Novagen), and grown in Luria-Bertani growth medium with 50 μg/ml of kanamycin. Protein induction methods were adapted from Goulas *et al* [27]. Cultures were grown at 37°C to an OD600_nm_ of 0.7 and protein production induced by 0.5 mm IPTG for 20 hours at 18°C. Cell pellets were collected then resuspended in 50 mM sodium phosphate pH 7.4, 300 mM NaCl with an EDTA-free Protease Inhibitor Cocktail (Roche Applied Science), and then lysed with a cell disrupter (Constant Cell Disruption Systems). Lysate was cleared by centrifugation at 35000 x g for 45 minutes, then mixed with cobalt resin (Talon-Clontech) previously equilibrated with 50 mM sodium phosphate pH 7.4, 300 mM NaCl and 10 mM imidazole. After overnight incubation at 4°C, His-tag rBFT proteins were eluted from the resin with 50 mM sodium phosphate pH 7.4, 300 mM NaCl and 150 mM imidazole. Mature CD (rBFT) was cleaved from the profragilysin with trypsin (Sigma) at ratio of 1:100 (w/w) for 3–4 hours at room temperature. The reaction was stopped by adding 5 mM AEBSF (4-(2-aminoethyl) benzenesulfonyl fluoride). The digested proteins were passed over a cobalt resin for second time to remove the N-terminus PD. The proteins were dialyzed overnight at 4°C against 20 mM Tris-HCl pH 7.4 and 1 mM DTT. The active rBFT metalloprotease was further purified on a MonoQ column followed by a size-exclusion chromatography with a Superdex 75 10/300. Purity of the proteins was assessed by SDS-PAGE. The recombinant proteins have the expected molecular weight of ~20 kDa. They were positively identified by N-terminal sequencing and LC/MS/MS.

### Generation of rabbit polyclonal antibodies against rBFT2 (L2-23)

Rabbit polyclonal antibody was produced by Cocalico Biological, Inc. (Reamtown, PA). Two New Zealand rabbits, each weighing around 3 kg, were immunized with rBFT2. Vaccination was performed using six doses, each injection as follows: a first dose containing 96 μg of the protein emulsified in 800 μl of Freund's complete adjuvant (CFA; Sigma) in a final total volume of 1 ml, while the subsequent doses contained 48 μg of emulsified protein with 400 μl of Freund's incomplete adjuvant (IFA; Sigma) in a final total volume of 1 ml. The injection was a combination of different route and multiple sites, with the initial inoculation done subcutaneously in the inguinal and axillary regions and intramuscular in the hind limb; boosts were subcutaneous along the back and intramuscular in hind limbs. Up to 0.2 ml per site for the injections were used. The last boost was on day 122 and the rabbits were sacrificed and exsanguinated on Day 139.

### Generation of mouse monoclonal antibodies against BFT isotypes

Mouse Monoclonals by Precision Antibody (A&G Pharmaceuticals). Briefly, monoclonal antibodies were developed by immunizing groups of 3 SJL/J mice with either rBFT1 or rBFT2 using Precision Antibody (Baltimore, MD) immunization protocols. Target-specific mouse antibody titers were determined by screening serial serum dilutions against immobilized rBFT1 and rBFT2 antigens in ELISAs. Once a sufficient titer was detected, a fusion was performed. B cells were harvested and fused with a murine myeloma partner. The fused cells were selected in HAT media and clones were derived from single cells. Culture supernatants were screened by ELISA against the rBFT1 or rBFT2. Specific hybridoma clones were expanded and tested for specificity in additional immunoassays.

### ELISA screening for anti-BFT mAbs

Hybridoma culture supernatants were initially screened by ELISA for murine IgG that bound recombinant rBFT1 or rBFT2. Nunc Maxisorp microtiter plates (Thermo Fisher Scientific) were coated with 100 μl/well of the antigen, diluted to 2 μg/ml in 1x PBS (pH 7.4) (Gibco), by overnight incubation at 4°C. Plates were blocked for 2 hr with 200 μl PBST (5% (w/v) dry milk/PBS/0.05% Tween 20) then incubated with culture supernatant, neat or diluted in PBS 1:2, for 1 hr at room temperature. After washing the plates four times with PBST, the wells were incubated with a 1:4000 dilution of horseradish peroxidase (HRP) conjugated goat anti-murine total IgG (Sigma) for 1 hr at rom temperature. Plates were again washed four times with PBST. The ELISA was developed for up to 15 min with 100 μl SureBlue TMB peroxidase substrate from KPL (San Jose, CA), and the final reactions were terminated by addition of 1 M HCl. Optical density (O.D.) measurements were obtained using a Biotek PowerWave HT at 450 nm. All experiments were performed in triplicates to obtain the mean values presented in figures.

### Isotype determination

The isotype of the mouse mAbs was determined using a Rapid ELISA Mouse mAb Isotyping Kit (ThermoFisher Scientific).

### Kinetics and affinity analysis using biacore surface plasmon resonance

Binding experiments were performed by Precision Antibody, using a Biacore 3000 (GE Healthcare) at 25°C. All 3 channels were coated with anti-mouse IgG Fc. Antibodies from the culture supernatants (0.2 μm filtered) were captured. BFT1 or BFT2 (100 nM) was then passed over the chip. Binding of antigen to the immobilized antibodies was monitored in real time to obtain on (ka) and off (kd) rates. The equilibrium constant (K_D_) was calculated from the observed ka and kd. The chip was regenerated (regeneration buffer: 10 mM glycine buffer, pH 1.75) to capture the next round of antibodies. This assay was performed on all clones. The assay buffer used was 10 mM HEPES buffer (pH 7.4), 150 mM NaCl, 3 mM EDTA, 0.005% P20 (polyoxyethylenesorbitan). The flow rate used for capturing the antigen was 10 ml/min. The flow rate for kinetics analysis was 30 ml/min. Chi square (χ^2^) analysis was carried out between the actual sensorgram and the sensorgram generated from the BIAnalysis software to determine the accuracy of the analysis. A χ^2^ value up to 2 was considered significant (accurate) and below 1 was highly significant (highly accurate).

### BFT1 epitope binning/antibody pairing

Octet Red96 (ForteBio) was used by Precision Antibody to identify antibodies binding to different epitopes on BFT toxin, by a label free Octet-based sandwich assay. Capture of the first mAb to saturation used immobilized goat anti-mouse Fc antibody on the biosensor. Then the rBFT1 antigen in solution was captured, followed by the detection of the binding of a second mAb to the target antigen.

### Purification of Abs

Rabbit polyclonal anti-BFT2 antibodies (L2-23) and mAbs from hybridoma culture supernatants were purified by affinity chromatography using a Protein G column (GE Healthcare). Briefly, a 1 ml Protein G column was equilibrated with 3 column volumes (CVs) of PBS. Hybridoma culture supernatant was adjusted to pH 7.0 by adding 10x PBS (Gibco) and then loaded on the column at 1 ml/min. IgG was eluted with 0.1 M glycine pH 2.0 and the pH of the eluate was adjusted to 7.0 with 0.5 M Tris (pH 9.0). The purified IgG was dialyzed against PBS overnight at 4°C, and the protein concentration quantified using Direct Detect (Millipore). Aliquots were stored at -20°C.

### Sandwich ELISA

Two types of sandwich ELISAs were developed to detect BFTs with either i) mAbs as capture antibody and rabbit polyclonal (L2-23) as detecting antibody or ii) mAbs as both capture and detecting antibodies. The rabbit antibody L2-23 generated against rBFT2 was determined to bind to both rBFT1 and rBFT2 by ELISA. For the first ELISA format using mAb as capture antibody and polyclonal antibody for detection, Nunc Maxisorb plates (Thermo Scientific) were coated with mAbs antibodies (10 μg/ml) in 1x PBS and incubated overnight at 4°C. Wells blocked with 200 μl of blocking solution (5% milk in PBST) for 1 hr at room temperature. After further washing, 100 μl of serially diluted BFTs are added and incubated at 1 hr at room temperature. Wells were washed four times with 200 μl of PBST prior to incubation with the detecting antibody L2-23 (10 μg/ml) for 1 hr at room temperature. Wells were washed four times with 200 μl of PBST before adding 100 μl of 1:4000 dilution of goat anti rabbit IgG conjugated to HRP (Sigma) and incubating for 40 minutes at room temperature. After washing with PBST, the ELISAs were developed with 100 μl/well of SureBlue TMB peroxidase substrate (KPL). Absorbance was read at OD 450 nm using a microtiter plate reader (Biotek). All samples were measured in triplicate. For the second format where mAbs were used as both capture and detecting antibodies, all the steps were identical, except detection Ab was biotinylated and reactions detected by StrepAvidin HRP (Pierce). In this second assay format, mAb 5A1 was used as the capture antibody while mAb 4G1 was used as the detecting antibody. Additional mAb:mAb pairs were tested (data not shown).

### Spiking and recovery experiment

Ten human stool samples from separate individuals were purchased from IVD Research, Inc, USA. PCR with primers for the *bft* genes was used to confirm BFT negative status of the stool samples. The stool filtrates were also negative for BFT in the cytotoxicity assay. Stool filtrates were prepared by first resuspending ~100 mg of stool in 1x PBS at a dilution of 1:4. The suspensions were then centrifuged at 2000 x g for 10 min followed by filtration through a 0.45 μm syringe filter (Millipore). The filtrats were used immediately or frozen at -20°C until required. Recovery was tested by spiking the stool filtrates with either purified rBFT1 or rBFT2 at different concentrations. The mean values, standard deviation (SD), and the coefficient of variation [(SD/mean)×100%], were calculated for each of these experiments.

### Cytotoxicity neutralization assay

The cytotoxicity assay for detecting *B*. *fragilis* enterotoxin rBFT1 and rBFT2 was performed as described previously Van Tassell *et al*, 1994 [26]. HT29/C1 cells (courtesy of Dr. Sears, Johns Hopkins University) were grown and maintained in DMEM (Gibco, Life Technologies, Inc., Grand Island, N.Y.) supplemented with penicillin (100 IU/ml), streptomycin (Sigma) (100 μg/ml) and 10% heat-inactivated fetal bovine serum (Hyclone Laboratories, Inc., Logan Utah) and human apotransferrin (Sigma), at 37°C under 5% CO_2_. For the cytotoxicity assay, the HT29/C1 cells were diluted to 1x10 ^4^ cells/ml and plated in 96-well plates tissue culture plates (200 μl/well) (Corning Glass Works, Corning, N.Y.). The cells were then allowed grow until they reached ~80% confluency. The cells were washed twice with DMEM without serum before addition of rBFTs. Either rBFT1 or rBFT2 (1 μg/well) was diluted in the DMEM growth medium and added to the cells. The plates were incubated at 37°C under 5% CO_2_ for 3 hours and then examined for the presence of characteristic toxin-induced cell morphological changes [28]. E-cadherin release was quantified by a commercially available ELISA kit (R&D Systems) and was performed according to manufacturer’s instructions. For the neutralization assay, 1 μg of either rBFT1 or rBFT2 and 10 μg of purified L2-23 or mAb IgG were incubated together in the 200 μl the DMEM growth medium for 30 minutes at room temperature before adding the HT29/C1 cells. Plates were read after 1–3 hours of incubation at 37°C under 5% CO_2_. Neutralization was indicated by a reduction in cytotoxic effect of at least 50%. Experiments were carried out in duplicate.

### Protease activity of rBFTs and its inhibition

Metalloprotease activities of rBFT1 and rBFT2 were assessed as described previously [27]. The protease activity was measured using the fluorescence-based EnzCheck assay kit containing BODIPY FL-casein (10 μg/ml) as a fluorescein conjugate (Invitrogen), and read on a microplate fluorimeter (FLx800, Biotek). All the reactions were carried out with 5 μg/ml of rBFT proteins in 10 mM Tris-HCl pH7.4, at room temperature. For the inhibition assay, 50 μg/ml antibodies were incubated with the toxin for 1 hr at room temperature, before the casein conjugate was added. Neutralization was indicated by a ≥ 50% reduction of protease activity compared to the control.

## Results

### Expression and purification of recombinant BFTs

**Fig 1:**
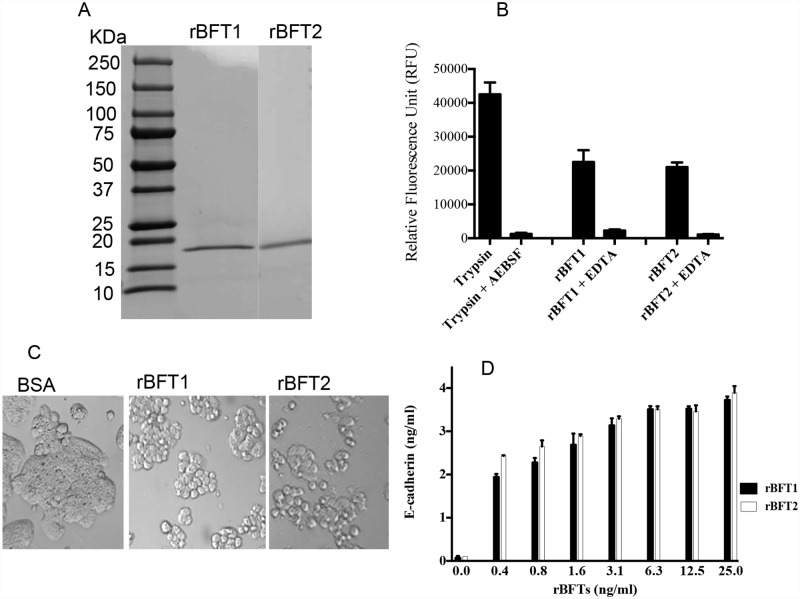
Biological activities of purified rBFT proteins. (A) The rBFT1 and rBFT2 proteins were expressed in *E*. *coli* as profragilysin-1 and profragilysin-2. The active, catalytic domains (rBFT) were cleaved from the prodomains with trypsin. The expected molecular weight of rBFT is ~20 kDa. The gel was stained with Coomassie blue. Purity of the proteins is estimated >90%. The cleaved rBFT proteins were confirmed by N-terminus sequencing. (B) Metalloprotease activities of rBFT1 and rBFT2 and their inhibition. rBFT1 and rBFT2 cleave casein and the protease activity is inhibited by an excess of the zinc-chelating agent, EDTA. Trypsin is used as a positive control protease. (C) Effect of rBFT proteins on the morphology HT29/C1 cells. Cells were treated with 10 μg/ml of either BSA or rBFT1 or rBFT2 for 3 h. The morphology of untreated cells was similar to the BSA-treated cells (data not shown). (D) Quantification of E-cadherin released into culture supernatants in the 3 h HT29/C1 cytotoxicity assay.

Recombinant BFT1 and BFT2 were expressed in *E*. *coli*. The catalytic domain was released by trypsin cleavage, purified (Fig 1A) and confirmed by N-terminal sequencing. The biological activity of the purified rBFTs was verified by two methods. First, rBFTs have an EDTA-sensitive proteolytic activity against casein (Fig 1B) that is consistent with BFTs being metalloproteases [27]. Second, the rBFTs were active in a cell-based cellular cytotoxicity assay. The rBFT1 and rBFT2 were active in this assay at ~0.4 and 2 ng/ml (Fig 1C), comparing favorably with native BFT1 and BFT2 purified from *B*. *fragilis* [29]. E-cadherin ectodomain released in the cellular cytotoxicity assay was quantified by ELISA (Fig 1D). Taken together, the results of the HT29/C1 assay and enzyme assay indicate that the purified rBFT proteins are biologically active.

### Mouse anti-BFT mAb development

**Fig 2:**
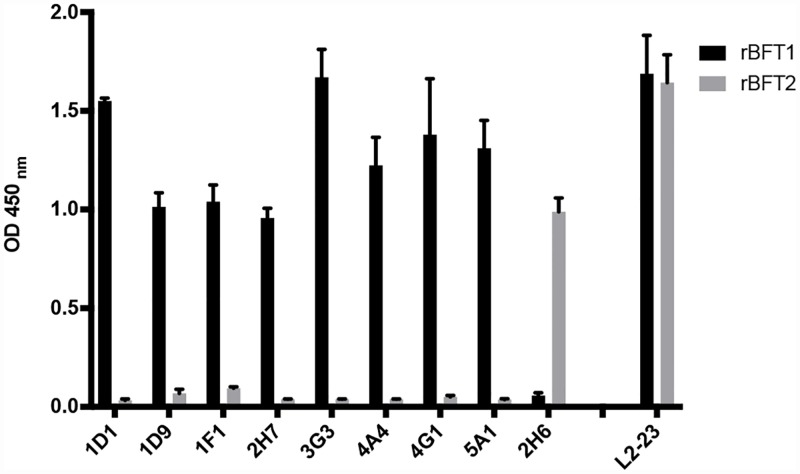
Mouse monoclonal antibodies specific to either rBFT1 or rBFT2. ELISA shows the binding of the different mAbs to wells coated with 2 μg/ml of either rBFT1 or rBFT2. All antibodies were used at a concentration of 10 μg/ml. Purified IgG from rabbit polyclonal antiserum generated against rBFT2 (L2-23) is shown as positive control for both BFT1 and BFT2.

Splenic cells from immunized mice with high anti-BFT1 or anti BFT2 antibody titers were fused with a mouse myeloma cell line. Hybridoma supernatants were analyzed by indirect ELISA against the rBFT proteins. Twenty-two stable hybridoma cell lines producing antibodies specific for either BFT1 or BFT2 were identified. Eight mAbs, designated as 1D1, 1D9, 1F1, 2H7, 3G3, 4A4, 4G1 and 5A1 were specific to rBFT1 and one mAb (2H6) reacted specifically with rBFT2 (Fig 2). The remaining 13 mAbs bound both rBFTs (data not shown). All mAbs were identified as IgG_1_ subclass with kappa light chains.

### Rabbit Polyclonal Antibody production (pAb)

The purified rabbit polyclonal antibody generated against rBFT2 (L2-23) bound with both rBFT1 and rBFT2 as demonstrated by ELISA (Fig 2). The rabbit polyclonal antibodies raised against rBFT2 reacted with both rBFT1 and rBFT2 by Western blot and ELISA (data not shown).

### Kinetics biacore

K_D_ and K_A_ were determined by Biacore Surface Plasmon Resonance (SPR) analysis. The results indicate that the mAbs have nanomolar affinities for rBFT1, with K_D_ values ranging from 5.42 x 10^−9^ M (3G3, 4A4) to 8.17 x 10^−12^ M (5A1) (Table 1). K_D_ for the lone rBFT2 specific mAb was determined to be 1.23 x 10^−9^. These values compare well with measured affinities for high-affinity mouse monoclonal antibodies, which typically have K_D_ values in the nanomolar range [30].

**Table 1:** Characterization of antibody binding activity.

Ligand	Analyte	ka (1/Ms)	kd (1/s)	Rmax (RU)	Conc of analyte	KA (1/M)	KD (M)
1D1	rBFT1	1.86X 10 ^5^	6.03X 10^−5^	36	100nM	3.09x 10^9^	3.24x10^-10^
1D9	rBFT1	2.08X 10^5^	2.06X 10^−5^	2.65	100nM	1.01x 10^10^	9.88x10^-11^
1F1	rBFT1	4.45X 10 ^5^	7.73X 10^−4^	57.7	100nM	5.76x 10^8^	1.74x10^-9^
2H7	rBFT1	1.99X 10 ^5^	8.28X 10−^4^	10.6	100nM	2.40x 10^8^	4.17x10^-9^
3G3	rBFT1	3.74X 10^5^	2.03X 10^−3^	9.43	100nM	1.84x 10^8^	5.42x10^-9^
4A4	rBFT1	1.59X 10 ^5^	8.61X 10^−4^	35.3	100nM	1.85x 10^8^	5.42x10^-9^
4G1	rBFT1	1.68X 10 ^5^	1.57X 10^−4^	14.5	100nM	1.06x 10^9^	9.40x10^-10^
5A1	rBFT1	3.62X 10^5^	2.96X 10^−6^	17.8	100nM	1.22x 10^11^	8.17x10^-12^
2H6	rBFT2	2.89X 10^5^	3.24X 10^−6^	49.9	100nM	8.13x 10^8^	1.23x10^-9^

### Epitope pairwise interaction analysis

In order to identify mAb pairs in a sandwich ELISA, each member of the pair of antibodies used in the assay must recognize a different epitope on the surface of the antigen. To determine whether any of the eight mAbs specific for rBFT1 share an epitope, a pairwise interaction analysis was undertaken using a ForteBio Octet RED96 interaction analysis reader. Antibody pairs were eliminated as potential pairs for use in a sandwich ELISA if the Octet analysis indicated that the candidate capture mAb prevents subsequent binding of the detection mAb. Not all possible pairs of the obtained mAbs were analyzed, but several candidate pairs were identified (data shown) as suitable for sandwich ELISA. Among these pairs, the pair with highest affinities were 5A1 (capture) and 4G1 (detecting).

### Sandwich ELISA

**Fig 3:**
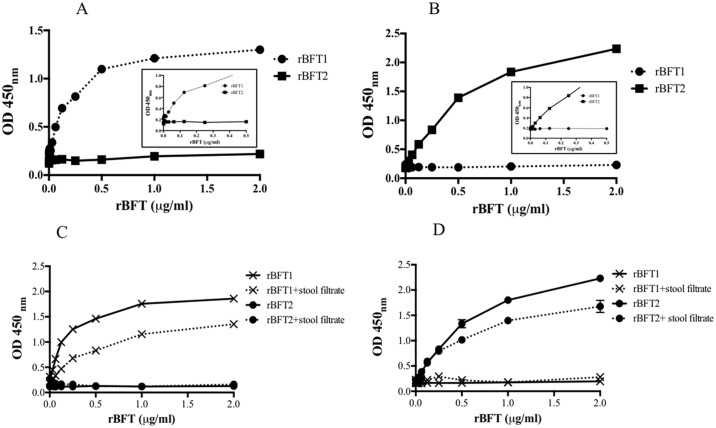
Sandwich ELISAs for isotype-specific detection of soluble rBFT1 and rBFT2 in buffer and in stool filtrate. (A) Microtiter plates were coated with 10 μg/ml of capture mAb 4G1 specific to rBFT1. Purified rBFT1 or rBFT2 were added to the plates and detected using rabbit polyclonal (pAb) antibody L2-23. Lower limit of detection was 20–40 ng/ml. (B) Microtiter plates were coated with 10 μg/ml of capture mAb 2H6 specific to rBFT2. Lower limit of detection was also 20–40 ng/ml. Detecting antibody was the rabbit polyclonal antibody L2-23. Each curve point represents the mean with ± standard deviation from three separate experiments. Stool filtrate components decrease detection sensitivity of the sandwich ELISA. (C) rBFT1 was serially diluted in PBS or in stool filtrate; (D) rBFT2 was serially diluted in PBS or in stool filtrate. For all stool filtrate ELISA, the average of ten samples are shown.

We used a sandwich ELISA to quantify BFT levels. Sandwich ELISAs are capable of detecting low-abundance antigens in complex, unpurified samples such as stool filtrate. The sandwich ELISA for the detection of BFT1 protein was developed using mAbs 3G3, 4G1 or 5A1 as the capture reagent and polyclonal antibody L2-23 as detecting antibody. Additionally an ELISA was developed with mAb 5A1 as capture and mAb 4G1 as detecting reagent. Standard curves for quantification of BFT1 toxin were generated with dilutions ranging from 0.002 to 2 μg/ml. Fig 3A shows the titration curve with an assay using mAb 4G1 as capture antibody and pAb L2-23 as detecting antibody. ELISA plates were coated with 100 μl of 10 μg/ml of 3G3, 4G1 or 5A1 before incubating the antigen and followed by the detecting with pAb L2-23. The detection limit for purified rBFT1 in standard reaction buffer was 20–40 ng/ml (Fig 3A). Similarly, a sandwich ELISA to detect BFT2 protein was performed with the one BFT2-specific monoclonal antibody available, 2H6 as the capture reagent and polyclonal rabbit antisera (L2-23) for detection. The BFT2 ELISA was also optimized to use 10 μg/ml of both capture mAb 2H6 and rabbit pAb L2-23 respectively (Fig 3B). The detection limit of purified rBFT2 standard reaction buffer was 20–40 ng/ml. The CV analyses for the intra- and inter-assay were 4.4% and 5.0% respectively, for both BFT1 and BFT2 ELISA with mAb as capture and pAb as detecting antibody.

### Spiking and recovery experiment

In order to simulate clinical stool samples, ten stool filtrates were spiked with multiple concentrations of BFT to determine the effect of the stool filtrate components on the sandwich ELISA. For BFT2, stool filtrate did not affect the limit of detection at low concentrations of fragilysin. For BFT1, the matrix of the stool filtrate reduced assay limit of detection about 50 percent to 60 ng/ml but the linearity and specificity of the assay were maintained (Fig 3C and 3D).

### Antibody neutralization of protease activity

**Fig 4:**
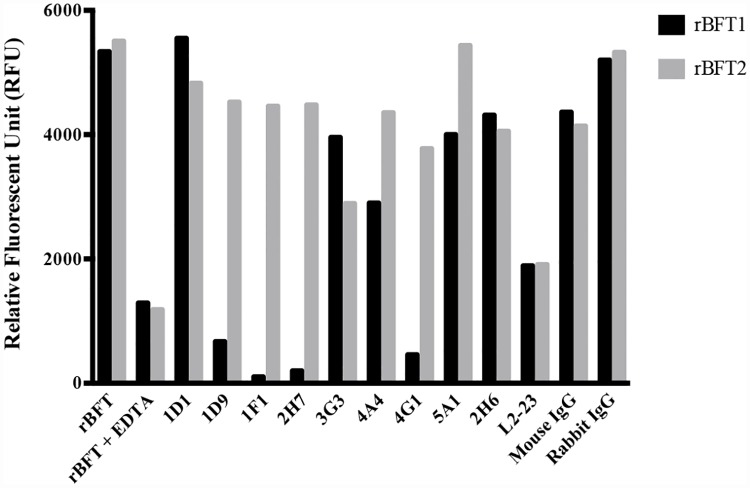
Casein assay to test the protease inhibition properties of the mAbs. 5 μg of each rBFT isotype protein were pre-incubated with an excess of each of the mAb before adding the casein reagents. mAbs 1D9, 1F1, 2H7, and 4G1, all specific to BFT1, significantly inhibit casein proteolysis by BFT1 but not BFT2. mAb 2H6, specific to BFT2, did not inhibit casein hydrolysis by BFT2.

We tested the ability of antibodies to inhibit the metalloprotease activity of rBFT1 and rBFT2 in a casein digestion assay (Fig 4). Antibodies were considered to be inhibitory if activity in the presence of the antibody was less than 50% of the uninhibited protease reaction. Polyclonal Ab L2-23 inhibited both rBFT1 and rBFT2 protease activities, as expected. Of the eight mAbs that specifically bind rBFT1, none inhibited rBFT2 protease activity and only 1D9, 1F1, 2H7, and 4G1 inhibited rBFT1 protease activity. The lone anti-rBFT2 mAb, 2H6, did not inhibit casein cleavage by rBFT2 or rBFT1.

### Antibody neutralization of cytotoxicity

**Fig 5:**
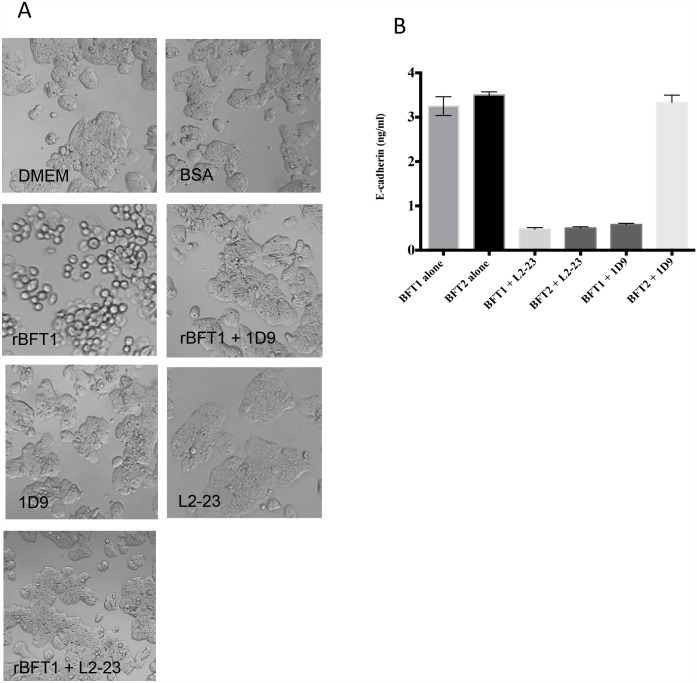
Neutralization of the cytotoxicity of rBFT1 by BFT1-specific mAb 1D9. (A) Microscopic images showing the effect of mAb 1D9 on rBFT1 exposed to HT29/C1 for 3 h at 37°C. The images were taken with a Zeiss Axiovert 40C at 400x magnification. Controls were DMEM alone, BSA or mAb 1D9 by itself. (B) E-cadherin release by HT29/C1 after exposure to rBFT1. HT29/C1 cells were exposed to rBFT1 for 3 h at 37°C and culture supernatant collected for E-cadherin measurements. The data are representative of three experiments.

The functional assay normally employed to detect native *B*. *fragilis* BFT is the HT29/C1 cell rounding assay. Briefly, this assay relies on the observation that HT29/C1 cells, a human colonic epithelial cell line, normally grows in culture as adherent cells, but in the presence of BFT become rounded as BFT induces endogenous E-cadherin cleavage [26, 27, 31]. To test whether any of the isolated mAbs are able to inhibit BFT-induced cell rounding, HT29/C1 cells were incubated with rBFT1 or rBFT2 in the presence of a mAb with affinity for that BFT isotype. The assay results were read after a 3 hour incubation. A positive result in the neutralization assay was indicated by reduction of HT29/C1 rounding by at least 50%. All 22 mAbs and the polyclonal antibodies were tested for their ability to inhibit the cytotoxic effect of rBFT1 and rBFT2. Only three antibodies inhibited rBFT cytotoxic activity: pAb L2-23; mAb 1D9, specific for rBFT1; and mAb 2H9, which binds both rBFT1 and rBFT2 (Fig 5A and data not shown). The neutralizing properties of mAb 1D9 and pAb L2-23 were also investigated by measuring the amount of E-cadherin released in the culture supernatant (Fig 5B). The amount of released of E-cadherin was considerably reduced in the presence of mAb 1D9 and pAb L2-23.

## Discussion

We have purified biologically active recombinant BFT1 and BFT2 as demonstrated by protease activity and ability to induce cytotoxic cell rounding. We used these two rBFTs to develop a unique set of high-affinity mAbs that are potentially useful for isotype-specific diagnostic identification of ETBF in clinical samples by sandwich ELISA. We have isolated eight monoclonal antibodies that are specific for BFT1, one that is specific for BFT2, and thirteen that are rBFT1 and rBFT2 reactive. A potential benefit of a BFT antibody panel is that mAbs can be used to standardize assays used worldwide in epidemiological studies of ETBF. We have developed two sandwich ELISAs: i) one using monoclonal antibodies for both capture and detection; ii) one using a monoclonal antibody for capture and a polyclonal antiserum for detection. In both cases the assays are specific and sensitive diagnostic tools capable of identifying rBFT in an isotype-specific manner in stool filtrate with a lower limit of detection of ~60 ng/ml or less.

For use in diagnostic assays, a monoclonal antibody must have high affinity, because the typical BFT concentration in the gut of affected individuals is likely to be low, though it has never been directly measured. The measured affinities of the mAbs produced after hybridoma isolation indicates that most of the mAbs have sufficient affinity for use in clinical diagnostic assays. A subset of these mAbs can be used to develop BFT assays for use with stool samples that are both sensitive and isotype-specific, and formatted in a manner amenable for high-throughput immunoassays. These assays are potentially important tools for analyzing the role of ETBF in IBD and CRC. Current methods for identification of ETBF are slow and require anaerobic culture of stool samples, usually in two stages, culminating with Bacteroides Bile Esculin (BBE) agar to culture *bacteroides* species from the many other species of bacteria present in stool samples. PCR must then be used to identify the presence of *B*. *fragilis*, and additional PCR must be performed to determine whether any *B*. *fragilis* is ETBF. A high-throughput, isotype-specific ELISA would improve in the rate of sample analysis and provide more information in a rapid immunoassay format.

Adding stool filtrate to the ELISAs resulted in increased rBFT limit of detection (approximately a 50% increase from a detection limit of ca. 40 ng/ml to 60 ng/ml), but demonstrated the feasibility of ELISAs detecting native BFTs in a matrix representative of the actual assay conditions likely to be encountered in a clinical setting. There is evidence [32] that lateral flow immunoassays may be less sensitive to contaminants or other factors in stool samples. A lateral flow stool assay using these mAbs could have a BFT limit of detection in the 20 to 40 ng/ml range, which was observed in ELISAs without added stool filtrate. Testing of confirmed ETBF-positive stool samples will be needed to determine if the limit of detection of the mAbs-based assays is sufficiently low to be useful in clinical settings. Of further interest will be to screen a large number of well-characterized and annotated human samples for the presence of BFT. It will then be possible to assess the utility of these ELISAs in a clinical setting by comparing the ELISA results with PCR and cell-rounding assay results. Obtaining and testing a sufficiently large bank of samples will be a priority for future studies.

There is no test to assess previous ETBF exposure (e.g., detection of serum anti-BFT IgG antibodies). Such a test could be of great benefit in tracking the disease progression of IBD or colorectal cancer, and would allow associating a history of ETBF infection with disease incidence and progression. A correlation may be important in determining the importance of ETBF as an etiologic agent in many diseases. An ELISA that can differentiate between BFT isotypes will be particularly valuable because there is currently no information associating disease severity with BFT toxin isotype. It is possible that some diseases will be more severe in one isotype or another, so it will be an important goal to assess patient populations with respect to various diseases (IBD, CRC, diarrhea) as well as BFT isotype. In recent studies it has been shown that patients with CRC have a higher incidence of *B*. *fragilis* that harbors the *bft* gene [20, 21]. The ultimate goal of correlating ETBF isotype with disease progression would be to identify ETBF as an etiological agent, in much the same way that the ability to diagnose exposure to *Helicobacter pylori* (Hp), by detection of specific serum anti-Hp antibodies and detection of gastric or fecal Hp, was critical to linking Hp to peptic ulcer disease and gastric cancer [33, 34].

Analysis of the toxin-antibody interactions, *e*.*g*., by BFT neutralizing epitope mapping, enables a better understanding of functional domains of the toxin molecules. We tested the ability of the mAbs to inhibit two biological functions of rBFT toxins: a) metalloprotease activity and b) cytotoxic activity. The HT29/C1 cell rounding and the casein cleavage assays identified two types of rBFT1 neutralizing mAbs: a) four that block casein cleavage by BFT1 and b) 1D9 that also neutralizes rBFT1 cytotoxicity. The anti-BFT2 mAb 2H9 is distinct in that it blocks rBFT2 cytotoxicity without inhibiting rBFT metalloprotease activity. These observations are unexpected as Franco *et al* [35] showed that a mutation in the zinc-binding domain adversely affected the cytotoxic property of BFT toxin, suggesting that the protease activity of BFTs is required for cell rounding. Our results indicate neutralization of protease activity is neither necessary nor sufficient to cause neutralization of BFT cytotoxicity. It is possible that in the case of mAbs that inhibit casein cleavage but not cell rounding, an epitope necessary for host cell binding was selected as an antibody target, thus preventing cell surface association. In this case, the protease activity will not be neutralized but the cytotoxic affects of the toxin will be reduced because the antibodies will interfere with the toxin’s ability to bind to target cells. The inhibition is an indication of whether the mAbs may be used for passive immunization, either on a continuing basis to treat chronic disease or in higher doses to treat acute infections.

In summary, ETBF is a known pathogen of humans worldwide, and has been implicated in the genesis and progression of diseases such as diarrhea, IBD, and CRC. The mAb-based BFT ELISAs developed in this study can potentially lead to a useful, non-invasive and rapid tool in the ever growing field to understand the relationship between gut flora and disease states, such as diabetes [36], depression [37, 38], and stress management among others [39, 40]. Current methods of identifying ETBF in clinical samples are time-consuming, do not directly detect BFT, and do not provide BFT isotype identification. The mAbs described here provide a potential pathway to a highly sensitive and isotype-specific assay, with the ability to develop into a much faster assay than is currently available in a high-throughput format. There are no data correlating severity of disease outcomes with BFT isotype, but the assays described here promise to provide a route forward in this area. Furthermore, mAb 1D9 inhibits both the cytotoxic and metalloprotease activities of BFT1, making it a potential candidate for use as a therapeutic lead for investigation of a passive immunization approach to treat patients with ETBF-associated disease. These new tools will enable screening of both symptomatic and asymptomatic patients for ETBF exposure (defined as fecal BFT) and will facilitate epidemiological studies to determine the prevalence of ETBF and its BFT isotypes. It is our hope that the field of gastroenterology will benefit by having these mAbs available as a tool for detection and prevention of this important pathogen.
